# Proteomics of the Dark-Ventral-Patch Sexual Signal in Male Red Deer

**DOI:** 10.3390/ani15020252

**Published:** 2025-01-17

**Authors:** Camilla Broggini, Paula V. Huertas-Abril, Alberto Membrillo, Eva de la Peña, Nieves Abril, Juan Carranza

**Affiliations:** 1 Wildlife Research Unit (UIRCP-UCO), University of Cordoba, 14014 Cordoba, Spain; b72depoa@uco.es (A.M.); evadelapenha@gmail.com (E.d.l.P.); jcarranza@uco.es (J.C.); 2 Department of Biochemistry and Molecular Biology, University of Cordoba, 14071 Cordoba, Spain; b52huabp@uco.es (P.V.H.-A.); bb1abdim@uco.es (N.A.); 3 UCD School of Agriculture & Food Science, University College Dublin, Belfield, D04 C1P1 Dublin, Ireland

**Keywords:** *Cervus elaphus*, chemical communication, shotgun proteomics, proteins

## Abstract

Sexual signals in animals encompass a variety of forms including visual, acoustic, and chemical signals that are fundamental for intra- and interspecific communication, including sexual selection processes. Among these, odor signals play a critical role. Chemical compounds involved in sexual signaling vary in nature, with lipids and proteins being particularly important. In the male Iberian red deer (*Cervus elaphus hispanicus*), the dark ventral patch has been identified as a key sexual signal during mating competition, associated with specific chemical compounds deposited during urination. Hair plays a significant role in this signaling due to its ability to retain compounds on the scales of the hair cuticle. In this study, we used a shotgun proteomic approach to investigate whether the ventral patch hair retains proteins on its surface that could reveal metabolic and cell-signaling adaptations potentially related to reproductive activity. Characterization of the origin and functionality of the proteins found in the hair of the dark ventral patch of male red deer, and their relationship to distinct metabolic pathways, provides an initial understanding of the potential role of these compounds in chemical communication in red deer intrasexual competition for mates.

## Simple Summary

In many animal species, including the Iberian red deer, odors play crucial roles in communication, especially during mating. In male red deer, a dark ventral patch, which is linked to urine markings, acts as a key signal in attracting mates and competing with other males. This patch is covered in hair that holds onto chemical compounds, which may offer insights into the animal’s health and reproductive readiness. This study aimed to explore whether proteins found on the hairs of the dark ventral patch could provide clues about the biological processes related to reproduction. We identified specific proteins on the hairs that might be involved in metabolic activities and cell signaling connected to mating. These findings help us understand how the chemical signals, carried by hair and urine might play a role in competition among males for mates. This research could be valuable in advancing our understanding of animal communication and sexual selection.

## 1. Introduction

Sexual signals are specific physical traits or behavioral displays showcased by animals to attract mates or to compete with rivals [1,2]. The development of sexual characteristics and the allocation of resources towards sexual traits play a fundamental role during sexual selection processes, including competition for mates, mate choice and all other forms of sexual interaction [1]. Sexual signals typically provide reliable information about an individual’s condition and social status [3]. The primary sexual signals of animals are generally classified as acoustic, visual or chemical, the first two groups being limited to daily activity [4,5].

In many mammalian species, chemical signaling plays a significant role in reproduction by conveying important information to receivers, such as the sender’s reproductive status or genetic quality [6,7,8]. Chemical signals manifest through an assortment of molecular and physiological processes and organs that are shaped by sexual selection [9]; moreover, sexual chemical signals are often produced via the sex-specific upregulation of biosynthetic enzymes [10]. Animals use a diverse selection of molecules to interact with mates and sexual rivals [11]. The chemical diversity of these traits varies from steroids to proteins, as well as differences in mixture constituents or proportions [12].

The Iberian red deer (*Cervus elaphus hispanicus* Hilzheimer, 1909) is a polygynous species, where males defend female harems or mating territories during the breeding season [13,14]. Male red deer manifest a diverse array of behaviors that demonstrate their fighting ability, dominance status and physical condition to attract females and deter rivals [15,16,17,18].

During the rutting season, male subjects urinate repeatedly on their ventral side, creating a dark spot. This area, known as the dark ventral patch, has been identified as a sexual signal in this species [19,20,21,22], and is present only during the breeding period. The size of this ventral patch increases during mating season, reaching lengths up to 70 cm [23], and it has been found to be linked to age and antler size, suggesting that it may serve as a reliable indicator of an individual’s reproductive effort during the mating season [24]. The dark pigmentation of the ventral region is attributed to the excretion of DOPEG (DL-3,4 dihydroxyphenyl glycol) in the urine, which results in the darkening of hair follicles in this area [23]. DOPEG, a metabolite of adrenaline, undergoes oxidation upon exposure to air, resulting in dark pigmentation [20].

Hairs in this ventral area may play a central role in sexual signaling. They are impregnated with strongly odoriferous compounds derived from glandular secretions, skin or urine, which have been shown to be related to individual features and the level of intrasexual competition in the population [19,21,23]. In addition to the lipidic compounds present in the patch [19,21], we expect a relevant role for the proteome in this area, given the significant functions of proteins in chemical signaling within sexual selection processes [25]. The study of the proteome of hair in the dark ventral patch of male Iberian red deer (*C. e. hispanicus*) can facilitate comprehension of the mechanisms of sexual signaling and communication. During the mating season, this patch functions not only as a visual cue but also as a chemical signal, with proteins and lipids potentially playing a significant role in mediating these signals [23]. Galván et al. [20] found that elevated levels of catecholamines in the urine of male deer correlate with increased pigmentation of the ventral patch, indicating that urinary metabolites are linked to the expression of this sexually selected trait. The specific proteins deposited in the hair of the ventral patch may serve as pheromonal cues, providing information about male health, genetic quality, and overall reproductive capability, which are critical factors in female mate choice. Moreover, compelling evidence increasingly supports the fascinating notion that secreted peptide or protein pheromones serve dual functions, acting not only as hormones within the body but also as chemosensory pheromones [25]. For instance, exocrine gland-secreting peptide 1 (ESP1) in mice and pheromone biosynthesis-activating neuropeptide (PBAN) in moths are two well-documented examples [26]. The structural properties of the hair in the dark ventral patch may enhance the dispersal of these protein chemical signals, facilitating olfactory communication and allowing males to effectively convey their fitness to potential mates and rivals [26]. This bimodal signaling, which combines visual and chemical cues, underscores the importance of studying proteins in the dark ventral patch to fully understand the dynamics of sexual selection in deer. In summary, investigating the proteome of the dark ventral patch is crucial for elucidating the complex interactions between visual and chemical signaling in the male red deer. Understanding how these proteins contribute to male competition and female choice can provide insight into the evolutionary pressures that shape these traits and their implications for reproductive success.

We have adopted a shotgun proteomic approach to investigate whether red deer ventral patch hairs retain proteins that allow us to infer the metabolic and cell-signaling adaptations that occur in male red deer to face intrasexual competition for mates. Shotgun proteomics has recently emerged as an effective method for characterizing proteomes in biological samples. The primary goal is to identify the form and quantity of each protein in a high-throughput manner by coupling liquid chromatography with tandem mass spectrometry [27]. Because females do not impregnate their abdomens with urine, it can be assumed that the comparison between males and females allows us to identify the differences in the protein compounds that make up this sexual signal in males, either coming directly from the urine or produced after specific processes in the impregnated area. We examined which compounds were exclusive to this area and which were shared between the ventral patch of males and the ventral area of females, with the objective of providing an initial understanding of the potential role of these compounds in chemical communication in male intrasexual competition for mates. The reasoning behind this approach was that the protein compounds on the hairs of this area may reflect an individual’s genomic sequence and expression levels, making them a potentially valuable source of information on genetic features, condition and even individual identity [28].

## 2. Materials and Methods

### 2.1. Study Site and Sample Collection

**Figure 1:**
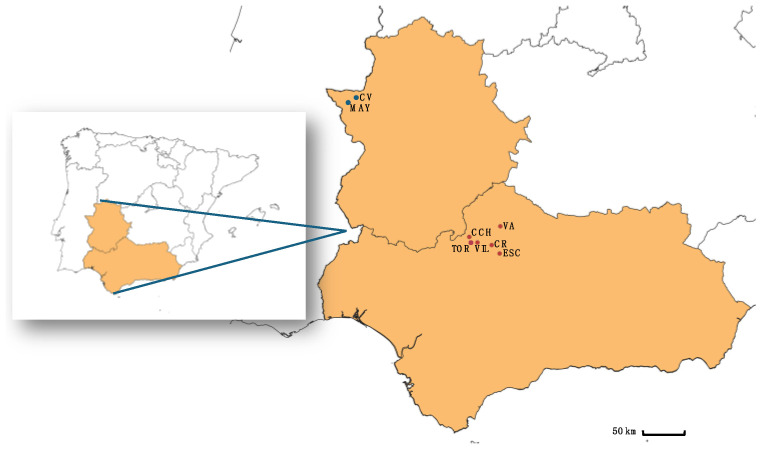
Sampling areas. The map highlights the geographical distribution of the hunting estates included in the study and located on the Iberian Peninsula: CCH, CR, ESC, TOR, VIL and VA in Andalusia, and CV and MAY in Extremadura, Spain.

This work comprised a total of 39 individuals, 18 females and 21 males of Iberian red deer (*C. e. hispanicus*), culled during the hunting season between October 2016 and February 2017. No animals were harvested specifically for the purpose of this study. We collected samples from carcasses of individuals hunted as part of legally regulated management or hunting activities, based on hunting plans approved by the autonomous governments of Andalusia and Extremadura (Spain). This is a non-invasive method that avoids the difficulties associated with capturing live animals to obtain samples [29]. The red deer specimens were collected from 6 hunting estates in the province of Córdoba (Andalusia, Spain) within the limits of Sierra Morena, located at distances ranging from 12 km to 42 km from each other, and 2 hunting farms in the province of Cáceres (Extremadura, Spain) within the limits of Sierra de San Pedro (Figure 1), located approximately 12 km apart.

The study areas are characterized by Mediterranean forest vegetation, primarily consisting of open dehesa, which features holm oaks (*Quercus* spp.) alongside Mediterranean shrubs and olive trees (*Olea europaea* L., 1753). Four of the hunting estates (CV, VA, CCH, ESC) were enclosed by 1.5 m high hunting fences, restricting the movement of individuals between different populations and resulting in high mating competition (HC). In contrast, the other four estates (MAY, TOR, CR, VIL) were open and lacked fencing, allowing for the free movement of individuals across hunting properties and characterized by low mating competition (LC). This distinction between fenced and open estates also results in differences in management practices, influencing the hunting pressure experienced by individuals [30].

Morphological measurements, including body size and thoracic perimeter, were recorded for each animal, regardless of sex, along with various measurements related to antler morphology and the length of the dark ventral patch in males. The age of the individuals was determined in the laboratory [31] by counting the cementum layers in the interradicular pad of the first molar of the lower jaw. All these data are included in Supplementary Table S1.

### 2.2. Hair Protein Isolation

Hair samples were collected from the ventral zone of male (dark ventral patch) and female (control) red deer. The hair was cut using clean scissors for each animal and the hair samples were directly transferred and preserved into clean glass vials, closed with a Teflon lid. The same procedure was used for the control vials, in which no hair was introduced, to determine the contaminants that could appear during handling in the environment, in the solvent or within the instruments later used in the laboratory. Hair samples were transferred to 2.5 mL Eppendorf tubes and urea (6–8 M) was added until it covered the hairs. The samples were left on a shaker at room temperature overnight. Afterwards, the samples were centrifuged, and the supernatant was transferred to a clean Eppendorf tube. This supernatant was then used to determine the hair proteome. The protein concentration in the supernatant was assessed using a Bradford assay [32], with bovine serum albumin as the standard.

### 2.3. Gel-Free LC-MS/MS Analysis

Protein extracts from each individual were cleared (one-dimensional SDS-10% polyacrylamide gel electrophoresis), reduced (20 mM dithiothreitol in 25 mM ammonium bicarbonate, 20 min, 55 °C), alkylated (40 mM iodoacetamide in 25 mM ammonium bicarbonate, in darkness, 20 min) and digested with trypsin (Promega, 2. 5 ng/μL in 25 mM ammonium bicarbonate, 37 °C, overnight) using standard protocols [33]. All analyses were performed at the proteomics unit of the Central Research Support Service (SCAI, University of Córdoba), using a Dionex Ultimate 3000 nano-UHPLC system (Thermo Fisher Scientific, Cork, Ireland) connected to an Orbitrap Fusion mass spectrometer (Thermo Fisher Scientific, Ireland) equipped with a nanoelectrospray ionization interface.

Digested samples were dried with SpeedVac and trapped on a 5 mm × 0.3 mm Acclaim Pepmap C18 precolumn (Thermo Fisher Scientific, Ireland) (2% acetonitrile/0.05% TFA, 5 min, 5 μL/min). The trapping column was in line with the separation column. Mobile phase buffers for peptide separation were composed of water/0.1% formic acid (buffer A) and 20% acetonitrile/0.1% formic acid (buffer B). The gradient was started at 40 °C, with a flow rate of 300 nL/min according to the following elution conditions: 4–35% buffer B for 60 min; 35–55% buffer B for 3 min; 55–90% buffer B for 3 min. The samples were then washed in buffer B (8 min) and re-equilibrated in 4% buffer B (15 min).

The nanoelectrospray ionization interface coupled to the UHLPC system converted the eluted peptides into gas-phase ions that were analyzed in an Orbitrap Fusion mass spectrometer (quadrupole–Orbitrap–quadrupole ion trap, Q-OT-qIT; Thermo Fisher Scientific, Ireland) operated in the positive mode. Peptides were detected in survey scans from 400 to 1500 *m*/*z*, with the Orbitrap resolution set at 120 K (at 200 *m*/*z*) and a 4 × 105 ion count target. Peptide precursors were detected in scanning ranges from 400 to 1500 *m*/*z*, with the Orbitrap resolution set at 120 K (at 200 *m*/*z*) and an ion counting target of 4 × 105. Tandem MS (MS2) spectra were acquired with an isolation window of 1.2 *m*/*z* using CID (collision-induced dissociation) fragmentation in the ion trap with 35% normalized collision energy. Monoisotopic precursor selection was turned on. The automatic gain control ion count target was set to 2 × 103 and the maximum ion injection time was 300 ms. Only precursor ions with charge states 2–5 were selected for MS2. Dynamic exclusion was set to 10 ppm tolerance around the selected precursor and its isotopes. The exclusion duration was 15 s.

Following MS2 data acquisition, raw data were processed for protein identification using MaxQuant v. 1.5.7.4 [34] without performing charge-state deconvolution or deisotoping. The search engine Andromeda [35] integrated into MaxQuant was used to interrogate the UniprotKB database either without any restrictions or restricted to *Cervus elaphus* (NCBI: txid9860). Proteins with no gene ID available for *C. elaphus* and those annotated as uncharacterized were replaced with the orthologues of *Cervidae*, *Bovidae* and other related species when an identity higher than 70% was present. MS2 spectra were searched, with a value of 10 ppm set for the mass tolerance of precursor ions. The fragment tolerance was set to 0.6 Da and a maximum of one missed cleavage was allowed. Cysteine carbamidomethylation was set as the fixed modification, and methionine oxidation was set as the variable modification. A target–decoy search strategy was used to estimate the false discovery rate (FDR). Peptide spectral matches were validated with a 1% FDR *q*-value. Peptide identifications were grouped into protein hits using a simple parsimony algorithm.

### 2.4. Label-Free Protein Quantification

For label-free protein quantification, we used Max-LFQ [36], part of the MaxQuant software suite, that applies retention time alignment and enables ’match between runs’ among replicates of each experimental condition to enhance identification. This algorithm combines and adjusts the peak areas obtained by the MaxQuant analysis of the ion chromatograms extracted from each peptide fragment around the predicted retention time into a protein intensity value. Perseus software v 1.5.6.0 [37] was used for downstream analyses to identify differentially expressed proteins. The resulting values are presented in Supplementary Table S2.

Data were normalized to the median, and LFQ-normalized intensity values were log_2_ transformed. Missing values were imputed using the lowest intensity value. The statistical significance of differences in protein abundance between females (control) and males was determined using a two-sample Student’s *t*-test with Benjamani–Hochberg correction [38], with an adjusted *p*-value < 0.05 set as the false discovery rate (FDR).

To characterize the proteome of abdominal hair in male individuals, we performed a functional analysis using the Search Tool for the Retrieval of Interacting Genes/Proteins (STRING, v. 11.0, https://string-db.org, accessed on 10 January 2024). STRING integrates all known and predicted protein–protein associations, including physical interactions and functional associations, to construct PPI (protein–protein interaction) networks with a minimum required interaction score of 0.4 [39]. Prior to analysis, protein accession numbers were converted into Gene IDs using the UniProt database conversion tool [40]. Furthermore, a Panther analysis (v. 18.0, released 1 August 2023; https://www.pantherdb.org/ (accessed on 12 January 2024).) was conducted when the information provided by the string analysis was not sufficient.

### 2.5. Statistical Analyses

We performed a MaxLFQ label-free quantification of the peptides detected in the nLC-MS2 analysis and used the Perseus software platform (https://maxquant.net/perseus/ (accessed on 12 March 2023)) [37] for statistical analysis. The statistical significance of protein abundance variations in male samples compared to the control (females) was determined using a two-sample Student’s *t*-test. The cut-off value was based on a Benjamani–Hochberg false discovery rate (FDR) of 5%. Prior to analysis, the data were normalized based on the median, and missing values were imputed using the value with the lowest intensity. Principal component analysis (PCA) was used to assess the relationships between proteins and their associations with males and females.

## 3. Results and Discussion

A shotgun proteomic method was implemented to investigate whether proteins retained in the ventral patch hairs of red deer could disclose the metabolic and cell-signaling adaptations that male red deer undergo to cope with intrasexual competition for mates.

**Figure 2:**
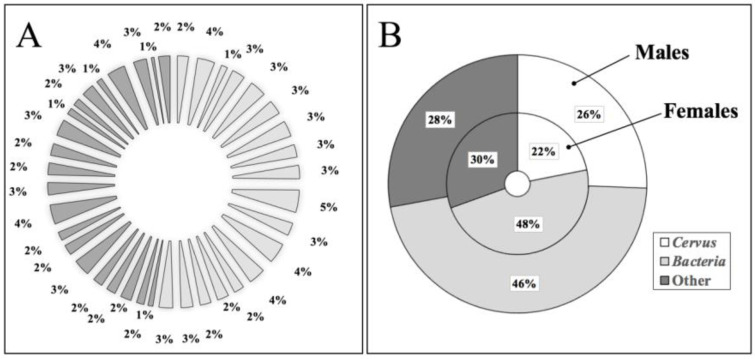
The red deer ventral proteome. (**A**) Contribution (%) of each red deer individual to the total identified protein counts. Clear gray was used for female samples (n = 18) and dark gray for male samples (n = 21); (**B**) major components of the red deer ventral proteomes.

We identified 17,951 proteins in total (Supplementary Table S3), 9387 in female samples and 8564 in male samples, with 2661 and 2796 unique proteins from females and males, respectively. Each individual, regardless of sex, contributed a similar percentage of proteins to the total amount identified (Figure 2A).

Most of the proteins (46–48%) were of bacterial origin, with only 22–26% corresponding to *Cervus* proteins (Supplementary Table S4; Figure 2B). Proteins classified as *Cervus* were those that matched the *C. elaphus* database. In addition, proteins lacking an available gene ID for *C. elaphus* or classified as uncharacterized were included in this category if they exhibited sequence identity greater than 70% with proteins from the *Cervidae*, *Bovidae* or other closely related families. The remaining 28–30% of proteins were identified as belonging to a wide range of species (*Homo*, rodents, plants, *algae*, amphibians, nematodes, etc.), which may inhabit the ecosystems where the red deer reside (Figure 2B and Supplementary Table S2).

### 3.1. Cervus Proteins Exclusive to Male Red Deer Dark Ventral Patch

**Figure 3:**
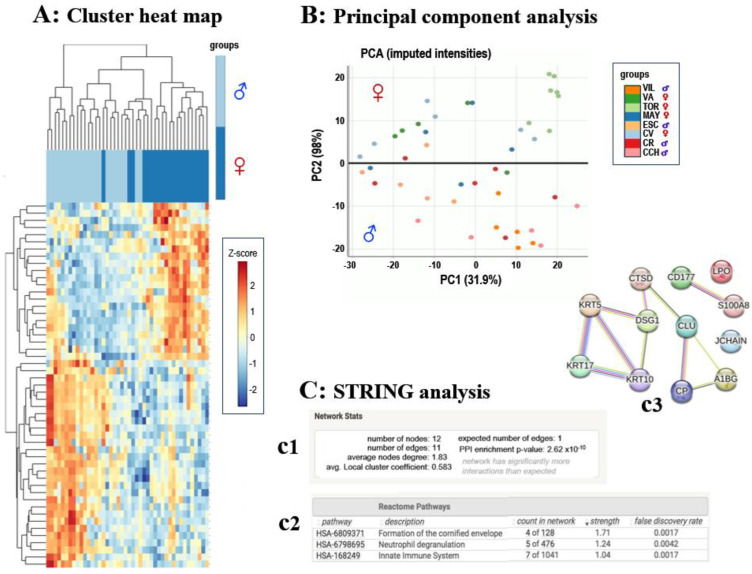
Differentially abundant proteins in the male red deer ventral dark patch. (**A**) A cluster heat map of proteomics profiles in males vs. control (females). Each row in the figure represents a protein and each column represents a sample (dark green: control samples; light green: male samples). The protein expression values are displayed on a color scale, with red indicating the highest upregulated levels and blue indicating the most downregulated levels. (**B**) Principal component analysis (PCA) plot representing proteomics data from the comparative analysis of control (female) and male groups. The plot displays two distinct clusters. (**C**) STRING analysis of proteomics data: (**c1**) Network statistics reporting data concerning the number of nodes and edges, the average node degree, the average local clustering coefficient, the expected number of edges and the protein–protein interaction (PPI) enrichment *p*-value. (**c2**) Reactome pathways analysis, showing the three most enriched pathways in the proteome of the male red deer ventral dark patch. (**c3**) Network interaction image showing nodes and edges between the proteins identified. The minimum required interaction score was 0.4 (medium confidence). The thickness of a line indicates the strength of the interaction between the proteins it connects.

To identify the proteins present in the male dark ventral patch that could reflect the systemic changes produced during the rutting season, we focused our analyses on the proteins of the Cervus group. Within this group, we found 51 proteins with statistically significant differences in their abundances when comparing between males and females. Differentially expressed proteins are shown in Supplementary Table S5 and in the heatmap in Figure 3A. Principal component analysis (PCA) showcases an almost complete separation of the two sexes along the PC2 axis based on the differences in the abundance of the 51 proteins between males and females (Figure 3B). However, most of these differentially expressed proteins were uncharacterized or not assigned to a specific protein species, so the functional analysis with the Reactome tool included in the STRING platform (https://string-db.org/ (accessed on 12 January 2024)) only showed an enrichment for males in proteins involved in the formation of the cornified envelope (DSG1 and keratins KRT 5, 10 and 17) and in immune response (CTSD, CD177, CLU, LPO, SA100A8, A1BG) (Figure 3). Keratins are proteins commonly found in the hair shaft proteome, so the higher abundance of some of these proteins in the ventral dark patch could be related to the hair specialization in this area [26]. On the other hand, sexual dimorphism affects a variety of immune processes [41], so the differences found in our results could potentially inform us about sex-specific immune responses.

In addition to the protein abundance analysis, we also conducted a presence/absence assessment to gain a broader understanding of the proteins in the dark ventral patch and the potential cellular processes occurring in rutting male red deer. The analysis of identified *Cervus* proteins (Supplementary Table S4) by Venn diagram (Venny v. 2.1, https://bioinfogp.cnb.csic.es/tools/venny/index.html (accessed on 1 February 2024)) suggested 392 common proteins, while 191 and 340 proteins were, respectively, female and male specific. The 340 proteins exclusive in the male ventral dark patch were analyzed with the STRING software v. 11.0. The protein–protein interaction (PPI) results included 238 nodes and 567 edges, in which nodes represented proteins and each edge represented a PPI relationship, with an average degree of 4.76 and a PPI enrichment value <1.00 × 10^−16^. Table 1 lists the main biological processes associated with the proteins occurring in the male dark ventral patch.

**Figure 4:**
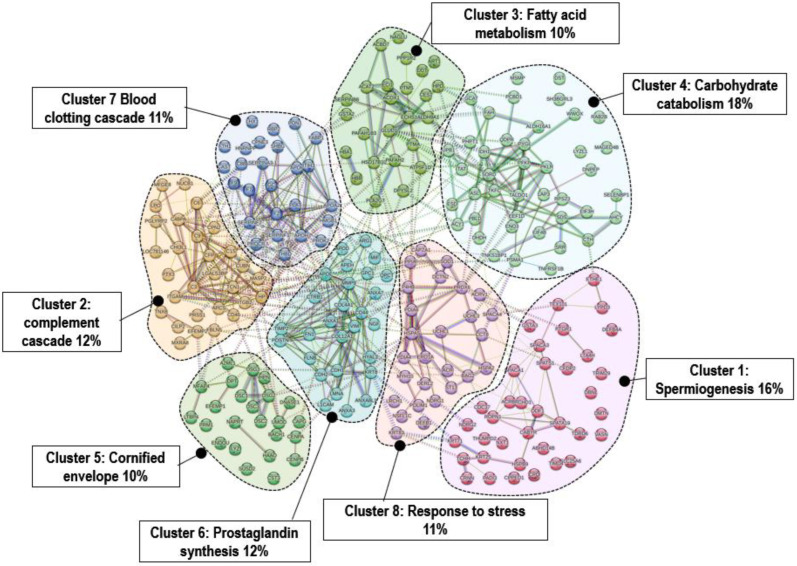
Functional analysis of protein–protein interaction (PPI): K-means clustering based on STRING database. Distribution of male dark ventral patch proteins among eight clusters suggested by STRING k-means analysis (default k-means clustering method).

Proteins usually are multifunctional and, hence, they can be annotated using two or more significantly different Gene Ontology (GO) terms that inform us of possible biological processes. For this reason, a K-Means algorithm was applied next to split the observations into homogeneous clusters in order to examine the similarities and dissimilarities between molecules within and between clusters. Eight clusters were clearly distinguished (Table 2, Figure 4), and the percentage of molecules in each cluster ranged from 10% to 18% (Figure 4).

The highlighting of proteins linked to spermiogenesis (*Cluster 1*) was not unexpected, as our reference group consisted of females and the ventral dark patch is routinely impregnated with urine and gland secretions. Similarly, the abundance of proteins related to “Cornified envelope” in *Cluster 5*, despite its label related to bovid horns, could also play a role in the seasonal regeneration of cervid antlers. Increased testosterone levels prior to the rutting period are associated with the formation of a densely structured antler cortex and an intense mineralization of the bone as well as the shedding of the specialized type of skin (velvet, with cornified epidermis) present in growing antlers [42].

Investment in sexual characters and reproductive effort, as well as unpredictable agonistic interactions, have been shown to result in costs in terms of physiological stress and parasite burden [43], in agreement with the immunocompetence handicap hypothesis [44]. It was also proposed for red deer that testosterone improves males’ reproductive investment and antler strength but imposes costs in terms of depressed parasite resistance [45]. *Cluster 2* is related to the complement cascade, a part of the innate immune defense that is made up of a large number of distinct plasma proteins that enhance the ability of antibodies and induce a series of inflammatory responses that help to fight infection. The complement system, a key sentinel of innate immunity, together with the coagulation system (*Cluster 7*), as a main actor in hemostasis, belong to the “first line of defense” against injurious stimuli and invaders [46]. It has been shown that thrombin, generated at inflammatory sites in response to complement activation, is a physiological agonist for the PKC-dependent pathway of decay accelerating factor (DAF) regulation in terms of a negative feedback loop preventing thrombosis during inflammation [47]. The complement activation product C5a has also been reported to induce tissue factor (TF) activity in human endothelial cells [48] and may activate the exogenous (TF-dependent) coagulation pathway.

*Cluster 4* is associated with carbohydrate catabolism, a primary process resulting in expeditious production. The mating season is an energetically costly period for males, as they must traverse vast distances in pursuit of females, defend harems or territories, engage in conflicts with other males and mate with females, all while operating at low feeding rates. This means that intense glycolytic activity is necessary to produce higher amount of energy (ATP), consequently justifying the finding of proteins such as ENO3, PFKP and IDH1, key regulatory enzymes in this pathway. The glycolysis pathway produces pyruvate, which is converted into acetyl coenzyme A and enters the Krebs cycle (KC). The KC is a central metabolic pathway utilized by many organisms for the catabolic degradation of carbohydrates, lipids and amino acids, which collaborates with the respiratory chain in the generation of ATP. However, the KC also serves as an important source of several intermediate compounds required for the synthesis of a diverse range of essential cellular molecules, including amino acids and fatty acids. During the rut, the red deer reduces its food intake but exhibits intensified activity and a notable acceleration of its metabolism. To compensate for the resulting energy deficit, the animal must utilize its body reserves, which may result in a loss of up to 25% of its body mass. Lipid reserve mobilization is probably the cause of this observed weight loss, as suggested by the deregulation of proteins linked to fatty acid metabolism (*Cluster 3*) such as acyl-CoA oxidase 1 (ACOX1) or enoyl-CoA hydratase, short chain 1 (ECHS1), the two first enzymes of the mitochondrial fatty acid beta-oxidation pathway. The protein 3-hydroxyacyl-CoA dehydrogenase type-2 (Hsd17b10) catalyzes the third step in the beta-oxidation cycle, but it is also involved in steroid metabolism, pointing to an increase in the synthesis of these lipid derivatives implicated in various functions associated with sexual reproduction. ACAT2 (acetyl-CoA C-acetyltransferase) is another protein linked to lipid metabolism that is present in the red deer ventral patch, suggesting that the liver is synthesizing ketone bodies as an alternative energy source in situations of near inanition. *Cluster 6* groups some proteins such as ANXA1, 3 (Annexins A1 and A3) and APOD (Apolipoprotein D), linked to the robust development of the antler [49] and mediators and regulators of the inflammatory processes. Inflammation and the intensified metabolism in the mating males probably induce a generalized stress response with the change in the abundance of proteins such as those included in the category “Cellular responses to stress” (*Cluster 8*). PRDX6 plays a role in cellular protection against oxidative stress by detoxifying peroxides and maintaining phospholipid homeostasis. SOD1 is responsible for the destruction of the radical superoxide ion, which is toxic to biological systems but is normally produced within cells. The presence of these proteins on the hair may serve as a reflection of the metabolic state of male red deer.

### 3.2. Possible Effect of the Level of Male–Male Competition

Previous studies have shown that the dark ventral patch is a good indicator of male willingness to invest in mating competition [24], demonstrating a positive correlation between the level of intrasexual competition caused by environmental conditions and the size of the ventral dark patch, with those individuals living in HC estates (i.e., high-pressure) having a bigger patch [23].

Here, it is shown that males have a characteristic proteome in the hairs of the ventral dark spot, reflecting an intense metabolism that enables them to cope with the mating effort. To gain a deeper understanding of the significance of the proteome involved in intrasexual competition for mates, we explored which of the 340 “male-only proteins” were expressed by males from high-competition populations (HC) and low-competition ones (LC). Supplementary Table S6 lists the 39 proteins that were exclusively present in male red deer sampled from HC populations and the 186 that were exclusively present in male red deer from LC populations.

**Figure 5:**
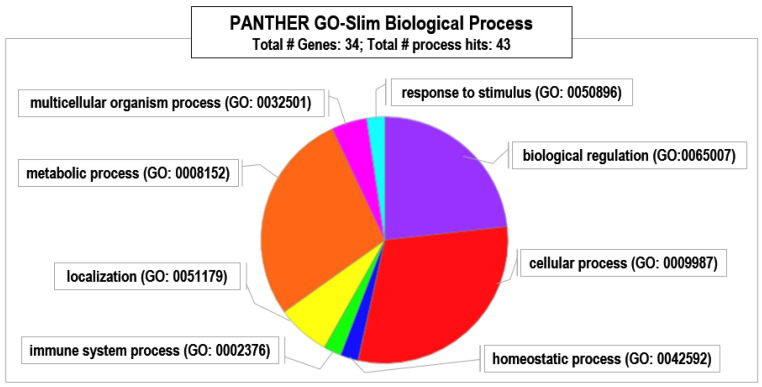
PANTHER Gene Ontology classification of proteins exclusively present in male red deer living in HC estates based on involvement in biological processes.

Panther analysis (Figure 5) suggested that males in HC populations experience increased regulation of cellular processes by specifically categorized proteins based on their biological roles. Additionally, the immune system and metabolic processes, such as the metabolism of amino acids and other nitrogenated compounds and the translational process, appeared reinforced.

**Figure 6:**
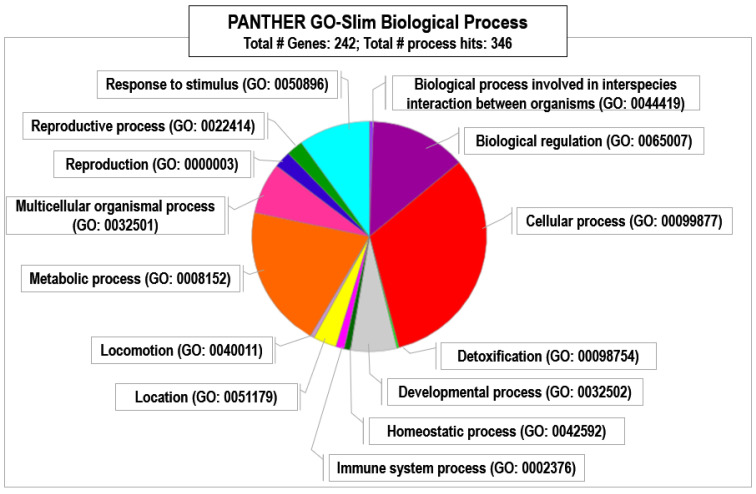
PANTHER classification of proteins exclusively present in male red deer living in LC estates.

For LC populations, in turn, we found exclusive proteins within six clusters related to different biological processes (Supplementary Table S6). Panther analyses (Figure 6) showed the relative abundance of proteins in each biological process category.

The pie chart displays that even though some of the most relevant metabolic and cellular processes are shared with HC male red deer, indicating that basic metabolic functions and cellular activities are important in both populations, other categories seem to be exclusively present in LC males like, for example, “response to stimulus” or “developmental process”, possibly indicating adaptation to their environments at the molecular level.

These results point to possible differences in the proteins present in males under high- and low-intrasexual-competition conditions, which could be related to previous results on the differences in the development of sex traits in these contrasting situations [21,23]. However, our exploration here must be considered preliminary, as we were not able to find statistically significant differences between sampled populations and our knowledge of the potential role of the identified proteins in those metabolic processes involved in the development and functions of sex traits and their consequences are still rudimentary.

### 3.3. Possible Redundancy of Sex Traits: Proteins Related to Antler Development

Based on the genes involved in antler development described in other studies [50], we explored which proteins occurred in the dark ventral patch. It was not so much a prospecting to see what we could find, but rather a more directed search for certain proteins that have been found to play a predominant role in antlers as a main sexual trait in red deer. Table S7 lists differentially abundant proteins in the dark ventral patch of males that are known to be related to antler development, distinguished between high- (HC) and low-competition (LC) populations.

Interestingly, the proteins found in male red deer living in HC populations and related to antler development were different from those we found in samples from LC populations (Supplementary Table S7). The proteins listed in the case of HC populations may suggest bone remodeling, tissue growth, cellular proliferation, and immune responses in antler growth. The presence of metabolic (APOA4) and bone-related proteins (COL1A1) indicates that these deer may be experiencing an environment that supports rapid, ongoing bone formation and healing. The tissue remodeling and repair processes indicated by proteins like CSTB and PLG might be linked to the high rate of bone turnover that occurs in antler growth.

Proteins found in red deer from LC populations, which likely have less-controlled environmental conditions allowing for more natural behaviors like movement and potential exposure to different stressors, suggest an adaptive response to more variable and possibly harsher environmental conditions. The presence of stress-response proteins (SOD1, PRDX6, HSPA5) could, for instance, indicate that these deer are experiencing more fluctuating conditions, which require enhanced mechanisms to cope with oxidative stress, maintain protein integrity, and regulate cellular processes. Furthermore, the fact that some of the proteins involved in antler growth may be found in the dark ventral patch as excretory products from urine is understandable. These smaller proteins, if present in the bloodstream, could be easily filtered by the kidneys depending on their abundance and potentially detected in the hair.

Since the dark ventral patch is a sexual signal, the presence in this area of proteins related to antlers suggests a potential meaning about antler characteristics also in this sexual signal. This presence could simply be a byproduct of the presence of specific metabolites in the urine or may have a role in communication as part of the meanings encompassed in the sexual signal. A number of studies have analyzed the possible role of redundancy in signaling and the presence of multiple signals that contribute to convincing the recipient [51,52,53,54,55,56]. The presence in the dark ventral patch of information related to antlers could represent a new case of redundancy or complementary signaling, which deserves further research.

## 4. Conclusions

This study is the first to explore the proteome profile of the dark ventral patch in the male Iberian red deer, a sexual signal shaped by intrasexual competition for mates. Our main goal was to describe the proteins present in the patch, with the aim of identifying proteins related to reproductive activity and sexual competition that could potentially be used in signaling. Through shotgun proteomic analysis, we identified specific proteins present in the ventral patch, many of which are associated with energy metabolism, immune response, and structural processes. Our findings indicate that proteins in the dark ventral patch of male Iberian red deer are not only structural components, but are also actively involved in energy metabolism, stress response, and immune functions. Proteins such as ACAT2, linked to lipid metabolism, suggest that during high reproductive effort, the liver may produce ketone bodies as an alternative energy source. This is crucial during the rutting season, when males face significant metabolic demands due to intense competition for mates, leading to stress and energy depletion. Additionally, immune-related proteins in the patch, such as those in the complement cascade, enhance antibody function and inflammatory responses, indicating an investment in immune defense during the breeding season. This aligns with the immunocompetence handicap hypothesis, which suggests that investment in sexual traits may come at a cost to immune function, thus necessitating a robust immune response. Moreover, the proteins present in the dark ventral patch may act as chemical signals that convey information about the health and genetic quality of males to potential mates. The dark ventral patch serves as both a visual and biochemical signal, highlighting the dual role of proteins in sexual selection and reproductive strategies. Future research should explore how variations in protein expression correlate with reproductive success and fitness in natural populations.

The differential proteome composition between males and females underscores the significance of patches in chemical communication, potentially conveying information about males’ health, reproductive effort, and dominance status. Additionally, the overlap of proteins linked to antler development suggests potential redundancy in sexual signals, wherein multiple traits may reinforce an individual’s competitive ability. These insights pave the way for future research into the molecular underpinnings of sexual selection and the interplay of visual and chemical signals in red deer.

In terms of practical applications for ecological management, our findings suggest that monitoring the proteostasis of the dark ventral patch could serve as a valuable tool for assessing population health and dynamics. Correlations between the size and condition of the ventral patch, male reproductive effort, and competitive ability suggest the potential for insights into the overall health of populations, particularly in environments that experience high levels of stress or competition. This approach could prove particularly useful in conservation efforts where understanding the reproductive strategies and health of populations is critical for effective management and preservation. In summary, the present study makes two contributions. First, it integrates biochemical insights into the understanding of sexual signaling, contributing to the theoretical framework of sexual selection. Second, this study offers practical implications for ecological management. By acknowledging the multifaceted nature of the dark ventral patch as an indicator of male quality, we can refine our methodologies for monitoring and managing Iberian red deer populations within their natural habitats.

## Figures and Tables

**Table 1:** Gene Ontology (GO) terms and associated biological processes identified after STRING analysis of proteins present in male dark ventral patch.

GO-Term	Description	Count in Network ^a^	Strength ^b^	FDR ^c^
Catabolism of aromatic amino acids				
GO:0006572	Tyrosine catabolic process	3/5	1.74	9.50 × 10^−03^
GO:0006559	L-phenylalanine catabolic process	4/8	1.66	1.40 × 10^−03^
GO:0009074	Aromatic amino acid family catabolic process	5/15	1.49	6.20 × 10^−04^
GO:0006570	Tyrosine metabolic process	4/13	1.45	4.60 × 10^−03^
GO:0009072	Aromatic amino acid family metabolic process	6/25	1.34	3.20 × 10^−04^
GO:0009063	Cellular amino acid catabolic process	10/101	0.96	1.50 × 10^−04^
GO:1901606	Alpha-amino acid catabolic process	10/86	1.03	4.32 × 10^−05^
GO:0008652	Cellular amino acid biosynthetic process	6/72	0.88	2.22 × 10^−02^
GO:1901605	Alpha-amino acid metabolic process	14/178	0.86	1.42 × 10^−05^
Catabolism of lipids				
GO:0034440	Lipid oxidation	6/78	0.85	3.00 × 10^−02^
GO:0060192	Negative regulation of lipase activity	3/9	1.49	2.62 × 10^−02^
GO:0006635	Fatty acid beta-oxidation	5/56	0.91	4.82 × 10^−02^
GO:0034374	Low-density lipoprotein particle remodeling	3/10	1.44	3.25 × 10^−02^
Catabolism of carbohydrates				
GO:0016052	Carbohydrate catabolic process	7/88	0.86	1.04 × 10^−02^
GO:0046365	Monosaccharide catabolic process	4/24	1.18	2.39 × 10^−02^
GO:0006090	Pyruvate metabolic process	5/57	0.91	4.94 × 10^−02^
GO:0046395	Carboxylic acid catabolic process	15/199	0.84	9.29 × 10^−06^
GO:0006026	Aminoglycan catabolic process	4/28	1.12	3.66 × 10^−02^
Response to stress				
GO:1990748	Cellular detoxification	9/107	0.89	1.30 × 10^−03^
GO:0098869	Cellular oxidant detoxification	8/94	0.89	2.80 × 10^−03^
GO:0042743	Hydrogen peroxide metabolic process	5/38	1.08	1.26 × 10^−02^
GO:0071456	Cellular response to hypoxia	6/73	0.88	2.33 × 10^−02^
GO:0051085	Chaperone cofactor-dependent protein refolding	4/30	1.09	4.53 × 10^−02^
GO:0006749	Glutathione metabolic process	5/56	0.91	4.82 × 10^−02^
GO:0019835	Cytolysis	5/25	1.26	3.00 × 10^−03^
Metabolism of structural proteins				
GO:0048251	Elastic fiber assembly	4/11	1.52	3.00 × 10^−03^
GO:0045104	Intermediate filament cytoskeleton organization	8/91	0.91	2.50 × 10^−03^
GO:0045109	Intermediate filament organization	6/75	0.87	2.60 × 10^−02^
GO:0010712	Regulation of collagen metabolic process	4/19	1.29	1.28 × 10^−02^
Complement activation				
GO:0006956	Complement activation	10/62	1.17	4.78 × 10^−06^
GO:0006958	Complement activation, classical pathway	8/43	1.23	3.54 × 10^−05^
GO:0006957	Complement activation, alternative pathway	4/11	1.52	3.00 × 10^−03^
Blood coagulation				
GO:0030194	Positive regulation of blood coagulation	4/15	1.39	6.70 × 10^−03^
GO:0050819	Negative regulation of coagulation	5/36	1.11	1.04 × 10^−02^
GO:0030193	Regulation of blood coagulation	6/50	1.04	4.80 × 10^−03^
GO:0050818	Regulation of coagulation	7/55	1.07	1.30 × 10^−03^
GO:0007596	Blood coagulation	9/100	0.92	8.60 × 10^−04^
GO:0090303	Positive regulation of wound healing	5/39	1.07	1.33 × 10^−02^
Immune response				
GO:0006953	Acute-phase response	4/21	1.24	1.64 × 10^−02^
GO:0002888	Positive regulation of myeloid leukocyte-mediated immunity	3/12	1.36	4.82 × 10^−02^

^a^ The first number indicates how many proteins in the network are annotated with a particular term. The second number indicates how many proteins in total (in the network and in the background) have this term assigned. ^b^ Log10 (observed/expected). This measure describes how large the enrichment effect is. It is the ratio between (i) the number of proteins in the network that are annotated with a term and (ii) the number of proteins that we expect to be annotated with this term in a random network of the same size. ^c^ False discovery rate. This measure describes how significant the enrichment is. Shown are *p*-values corrected for multiple testing within each category using the Benjamini–Hochberg procedure.

**Table 2:** K-means clustering of proteins present in male dark ventral patch based on STRING database.

Cluster ID	Gene Count	Network Stats (Number of Nodes; Average Node Degree; Avg. Local Clustering Coefficient; Expected Number of Edges) and PPI Enrichment *p*-Value	Biological Process	Protein Names
Cluster 1	37	37; 29; 1.57; 0.362; 1 <1.0 × 10^−16^	Spermiogenesis. Acrosome biogenesis	ABHD14B, ACRBP, CABYR, CACHD1, CDC37, CFDP2, CPPED1, CPQ, CRNN, DBNL, DEFB4A, DMTN, ETHE1, GSTA3, HSPB9, KRT25, KRT71, LTA4H, LYPD3, NDRG2, NXT1, ODF1, PADI3, PTGR1, ROPN1, SLC25A6, SPACA1, SPACA3, SPATA19, SPATS1, TCHH, TDRD6, TEX101, TIMD4, TTHUMPD2, RIM29, VASN
Cluster 2	29	29; 50; 3.45; 0.529; 1 <1.0 × 10^−16^	Complement cascades	APCS, C1S, C3, C4BPA, C6, C9, CD46, CFP, CHI3L1, CILP2, CPN2, CUBN, EFEMP2, FBLN5, HP, ITGAM, ITGB2, LGALS3BP, LOC781146, LPO, TNX, MASP2, MFGE8, MXRA8, NUCB1, PGLYRP2, PRSS1, PTX3, TCN1, TNXB
Cluster 3	25	25; 29; 2.9; 0.397; 2 <1.0 × 10^−16^	Fatty acid metabolism	ACAT2, ACBD7, ACOX1, ALDH9A1, ART1, ATP5F1D, CES1, DDT, DPYSL2, ECHS1, ECI1, GLUD1, GSTA2, HBA1, HBB, HPD, HSD17B10, NAGLU, PAFAH1B3, PAFAH2, PLA2G7, PPP1R2, PTMA, PTMS, SERPINB6
Cluster 4	42	42; 61; 2.32; 0.679; 9 <1.0 × 10^−16^	Carbohydrate catabolism	ACY1, AHCY, ALDH16A1, ASL, CTH, DHDH, DNPEP, DST, EEF1D, EIF3H, EIF4B, ENO3, ESD, FAH, GCAT, IDH1, LAP3, LYZL1, MAGED4B, MSMP, PBLD, PCBD1, PFKP, PHPT1, PKLR, PSMA1, PYGL, QDPR, RAB2B, RPS23, SDS, SELENBP1, SH3BGRL3, SORD, SRR, TALDO1, TAT, TKFC, TNFRSF1B, TNKS1BP1, UPB1, WWOX
Cluster 5	24	24; 15; 1.25; 0.533; 8 <1.0 × 10^−16^	Cornified envelope	A2ML1, BACH1, CAPG, CENPA, CENPB, CST3, DNASE1, DPT, DSC1, DSC2, DSC3, DSG2, DSG3, EFEMP1, ENDOU, HAAO, HPN, LTBP4, LYZ, MFAP4, NAPRT, PRM1, SUSD2, UMOD
Cluster 6	28	28; 82; 5.86; 0.714 <1.0 × 10^−16^	Signaling and regulation	ANXA1, ANXA3, ANXA7, ANXA8L1, APEH, APOD, APOE, ARG1, CD44, CDH1, CDH2, COL12A1, COL4A1, CTRB1, FLNB, FN1, GPC1, HYAL3, KRT8, L1CAM, LMNA, MIF, MMP9, NGF, POSTN, QPCT, TIMP2, VIM
Cluster 7	26	26; 47; 3.62; 0.636; 1 <1.0 × 10^−16^	Blood clotting cascade	APOA2, APOF, APOH, C8B, CAST, CPNE3, F2, F7, F9, FABP1, FTH1, GC, HMGB1, HNRNPF, ITIH3, LHX1, PLAT, PON1, PROCR, PROK2, RBP2, SERPINA3-1, SERPINF1, SERPINF2, SHBG, THBD
Cluster 8	27	27; 35; 2.59; 0.647; 3 <1.0 × 10^−16^	Cellular responses to stress	ACR, ACRV1, BAG2, CAPZA1, CCT8, DCTN2, DEFB1, DERL2, ERO1A, HSPA2, HSPA5, KRT83, LRCH1, MYH10, NDRG1, NSFL1C, P4HB, PDIA4, PDIA6, PDLIM1, PPIA, PRDX6, SOD1, SPACA4, ST13, UCHL1, UCHL3

## Data Availability

The original contributions presented in this study are included in the article/Supplementary Material. Further inquiries can be directed to the corresponding author.

## Supplementary Materials

The following supporting information can be downloaded at: https://www.mdpi.com/article/10.3390/ani15020252/s1, Table S1: Summary of morphometric data from red deer (*Cervus elaphus hispanicus*) study animals, including individuals and their populations of origin; Table S2: Total proteins identified in the ventral area of the male and female subjects under study; Table S3: Total proteins identified; Table S4: Protein species (unique) identified in the belly ’ecosystem’ of female and male red deer; Table S5: Differentially expressed proteins in red deer male ventral dark patch; Table S6: STRING mapping of proteins exclusively present in male red deer living in fenced and unfenced estates; Table S7: List of differential abundant proteins of antler samples that we exclusively found in dark ventral patches of male red deer living in fenced and unfenced estates.
