# Supplementary material for: Proteomics of the Dark-Ventral-Patch Sexual Signal in Male Red Deer
Source: Animals (Basel). 2025 Jan 17;15(2):252. doi: 10.3390/ani15020252 (PMC11758638; doi:10.3390/ani15020252)
Supplement: Supplementary file 1 [file animals-15-00252-s001.zip › Table S7 - List of differential abundant proteins of the antler samples of red deer.pdf]

Suppl. Table S7

List of differential abundant proteins of the antler samples that we **exclusively** found **in** the dark ventral patch of **male red deer living in <u>fenced</u> estates**.

String Id	Preferred Name	Annotation
9913.ENSBTAP00000001674	PLG	Plasmin heavy chain A, short form; Plasmin dissolves the fibrin of blood clots and acts as a proteolytic factor in a variety of other processes including embryonic development, tissue remodeling, tumor invasion, and inflammation. In ovulation, weakens the walls of the Graafian follicle. It activates the urokinase-type plasminogen activator, collagenases and several complement zymogens, such as C1 and C5. Cleavage of fibronectin and laminin leads to cell detachment and apoptosis. Also cleaves fibrin, thrombospondin and von Willebrand factor. Role in tissue remodeling.
9913.ENSBTAP00000023309	FETUB	Fetuin-B; Protease inhibitor required for egg fertilization. Required to prevent premature zona pellucida hardening before fertilization, probably by inhibiting the protease activity of ASTL, a protease that mediates the cleavage of ZP2 and triggers zona pellucida hardening (By similarity).
9913.ENSBTAP00000026345	APOA4	Apolipoprotein A-IV; May have a role in chylomicrons and VLDL secretion and catabolism. Required for efficient activation of lipoprotein lipase by ApoC-II; potent activator of LCAT. Apoa-IV is a major component of HDL and chylomicrons (By similarity).
9913.ENSBTAP00000017420	COL1A1	Collagen alpha-1(I) chain; Type I collagen is a member of group I collagen (fibrillar forming collagen).
9913.ENSBTAP00000069446	A1BG	Alpha-1B-glycoprotein.
9913.ENSBTAP00000000683	CSTB	Cystatin-B; This is an intracellular thiol proteinase inhibitor.
9913.ENSBTAP00000015716	EEF1G	Elongation factor 1-gamma; Probably plays a role in anchoring the complex to other cellular components.
9913.ENSBTAP00000009469	TAGLN	Transgelin; Actin cross-linking/gelling protein.
9913.ENSBTAP00000005581	EEF2	Elongation factor 2; Catalyzes the GTP-dependent ribosomal translocation step during translation elongation. During this step, the ribosome changes from the pre-translocational (PRE) to the post-translocational (POST) state as the newly formed A-site-bound peptidyl-tRNA and P-site-bound deacylated tRNA move to the P and E sites, respectively. Catalyzes the coordinated movement of the two tRNA molecules, the mRNA and conformational changes in the ribosome (By similarity); Belongs to the TRAFAC class translation factor GTPase superfamily. Classic translation factor GTPase family. EF-G/EF [...]
9913.ENSBTAP00000066720	PTBP1	Polypyrimidine tract-binding protein 1; Plays a role in pre-mRNA splicing and in the regulation of alternative splicing events. Activates exon skipping of its own pre- mRNA during muscle cell differentiation. Binds to the polypyrimidine tract of introns. May promote RNA looping when bound to two separate polypyrimidine tracts in the same pre-mRNA. May promote the binding of U2 snRNP to pre-mRNA. Cooperates with RAVER1 to modulate switching between mutually exclusive exons during maturation of the TPM1 pre- mRNA. Represses the splicing of MAPT/Tau exon 10 (By similarity).

List of differential abundant proteins of the antler samples, that we **exclusively** found **in male red deer living in <u>unfenced</u> estates** (STRING mapping of the proteins exclusively present in male red deer living in unfenced estates).

String Id	Preferred Name	Annotation
9913.ENSBTAP00000024092	**AHCY**	Adenosylhomocysteinase; Adenosylhomocysteine is a competitive inhibitor of S- adenosyl-L-methionine-dependent methyl transferase reactions; therefore adenosylhomocysteinase may play a key role in the control of methylations via regulation of the intracellular concentration of adenosylhomocysteine.
9913.ENSBTAP00000032384	**SOD1**	Superoxide dismutase [Cu-Zn]; Destroys radicals which are normally produced within the cells and which are toxic to biological systems. Belongs to the Cu-Zn superoxide dismutase family.
9913.ENSBTAP00000006383	**PRDX6**	Peroxiredoxin-6; Thiol-specific peroxidase that catalyzes the reduction of hydrogen peroxide and organic hydroperoxides to water and alcohols, respectively. Can reduce H(2)O(2) and short chain organic, fatty acid, and phospholipid hydroperoxides. Also has phospholipase activity, and can therefore either reduce the oxidized sn-2 fatty acyl grup of phospholipids (peroxidase activity) or hydrolyze the sn-2 ester bond of phospholipids (phospholipase activity). These activities are dependent on binding to phospholipids at acidic pH and to oxidized phospholipds at cytosolic pH. Plays a role [...]
9913.ENSBTAP00000052422	**HSPA5**	Endoplasmic reticulum chaperone BiP; Endoplasmic reticulum chaperone that plays a key role in protein folding and quality control in the endoplasmic reticulum lumen (By similarity). Involved in the correct folding of proteins and degradation of misfolded proteins via its interaction with DNAJC10/ERdj5, probably to facilitate the release of DNAJC10/ERdj5 from its substrate (By similarity). Acts as a key repressor of the ERN1/IRE1-mediated unfolded protein response (UPR). In the unstressed endoplasmic reticulum, recruited by DNAJB9/ERdj4 to the luminal region of ERN1/IRE1, leading to dis [...]
9913.ENSBTAP00000002508	**PDIA6**	Protein disulfide isomerase family A member 6; Belongs to the protein disulfide isomerase family.
9913.ENSBTAP00000019499	**EEF1D**	Elongation factor 1-delta; EF-1-beta and EF-1-delta stimulate the exchange of GDP bound to EF-1-alpha to GTP; Belongs to the EF-1-beta/EF-1-delta family.
9913.ENSBTAP00000022782	**PDIA4**	Protein disulfide-isomerase A4.
9913.ENSBTAP00000072070	**ST13**	ST13 Hsp70 interacting protein.
9913.ENSBTAP00000024572	**VIM**	Vimentin; Vimentins are class-III intermediate filaments found in various non-epithelial cells, especially mesenchymal cells. Vimentin is attached to the nucleus, endoplasmic reticulum, and mitochondria, either laterally or terminally.
